# Supplementary figures and images for: A Grad-seq View of RNA and Protein Complexes in Pseudomonas aeruginosa under Standard and Bacteriophage Predation Conditions
Source: mBio. 2021 Feb 9;12(1):e03454-20. doi: 10.1128/mBio.03454-20 (PMC8545117; doi:10.1128/mBio.03454-20)

Figure S1

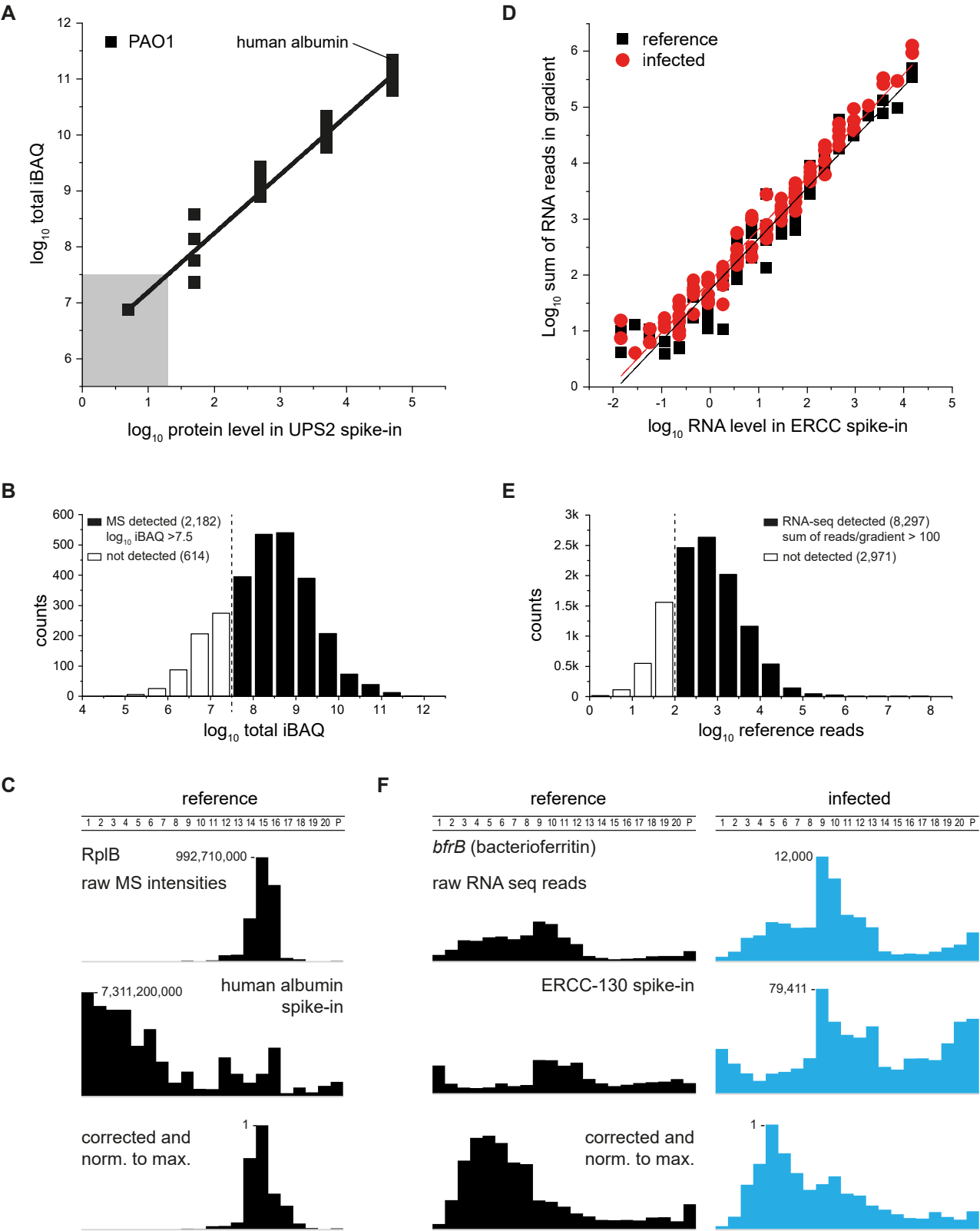

Supplement: FIG S1 [file mbio.03454-20-sf001.pdf]

Figure S2

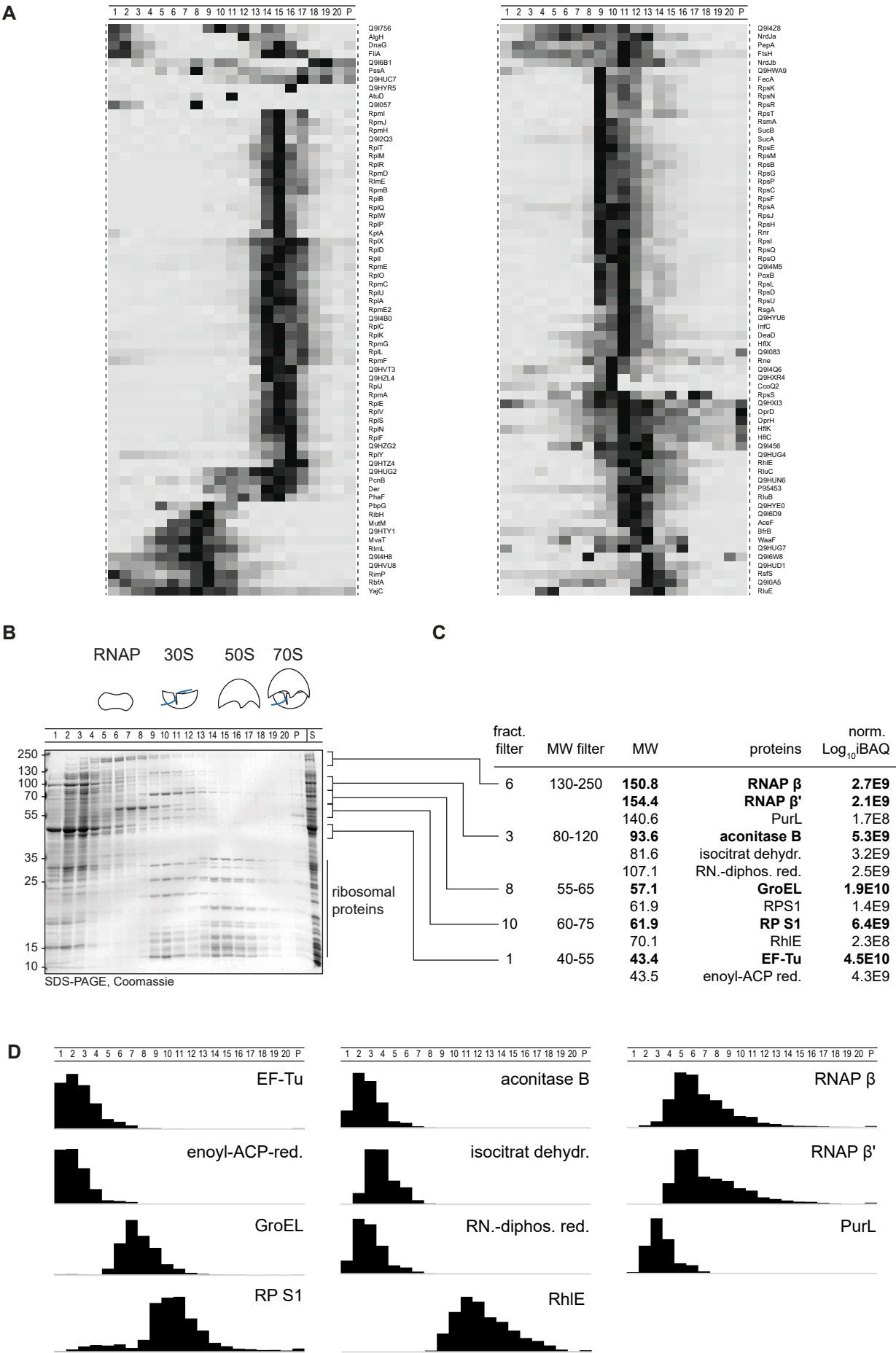

Supplement: FIG S2 [file mbio.03454-20-sf002.pdf]

Figure S3

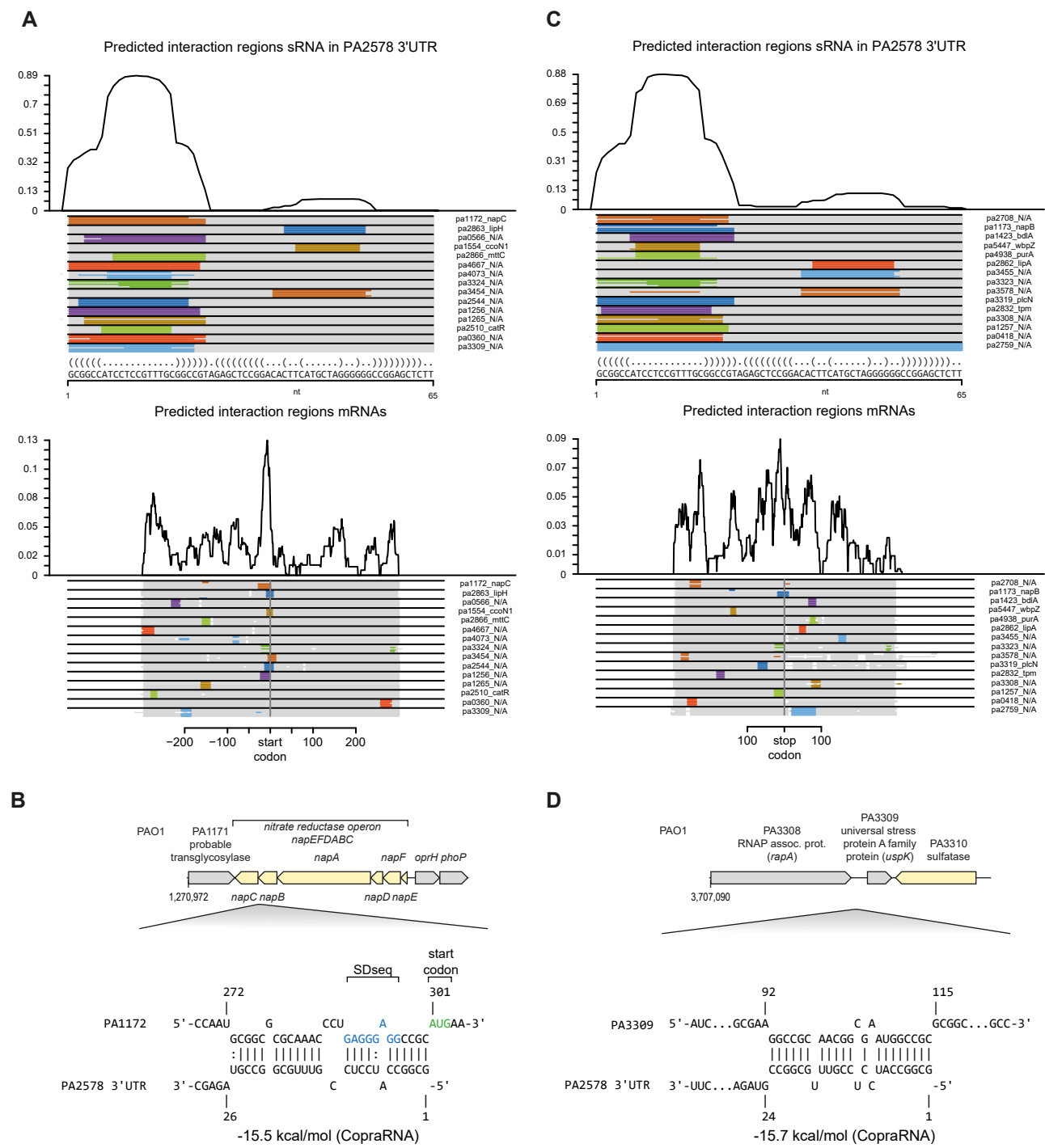

Supplement: FIG S3 [file mbio.03454-20-sf003.pdf]

Figure S4

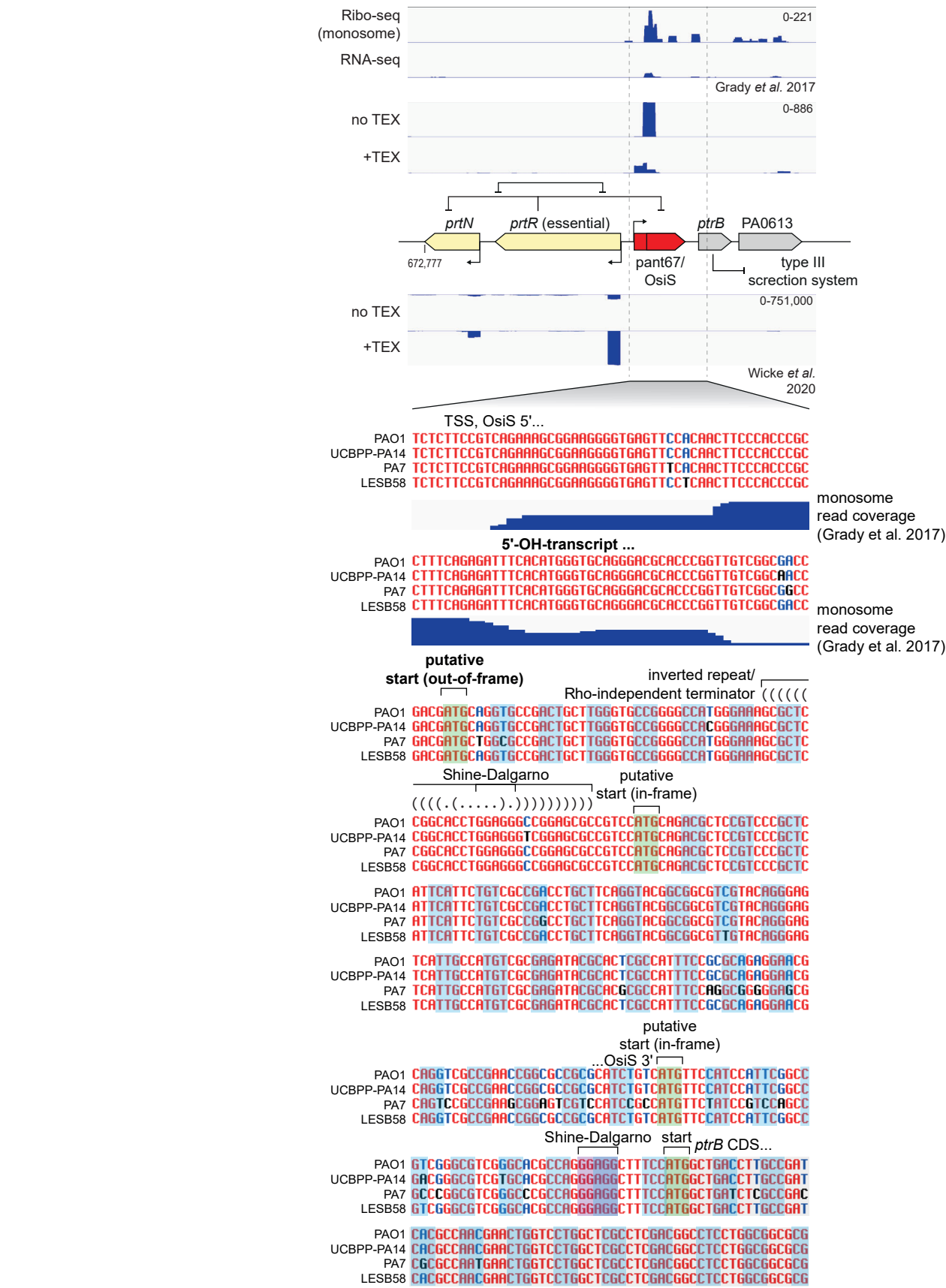

Supplement: FIG S4 [file mbio.03454-20-sf004.pdf]

Figure S5

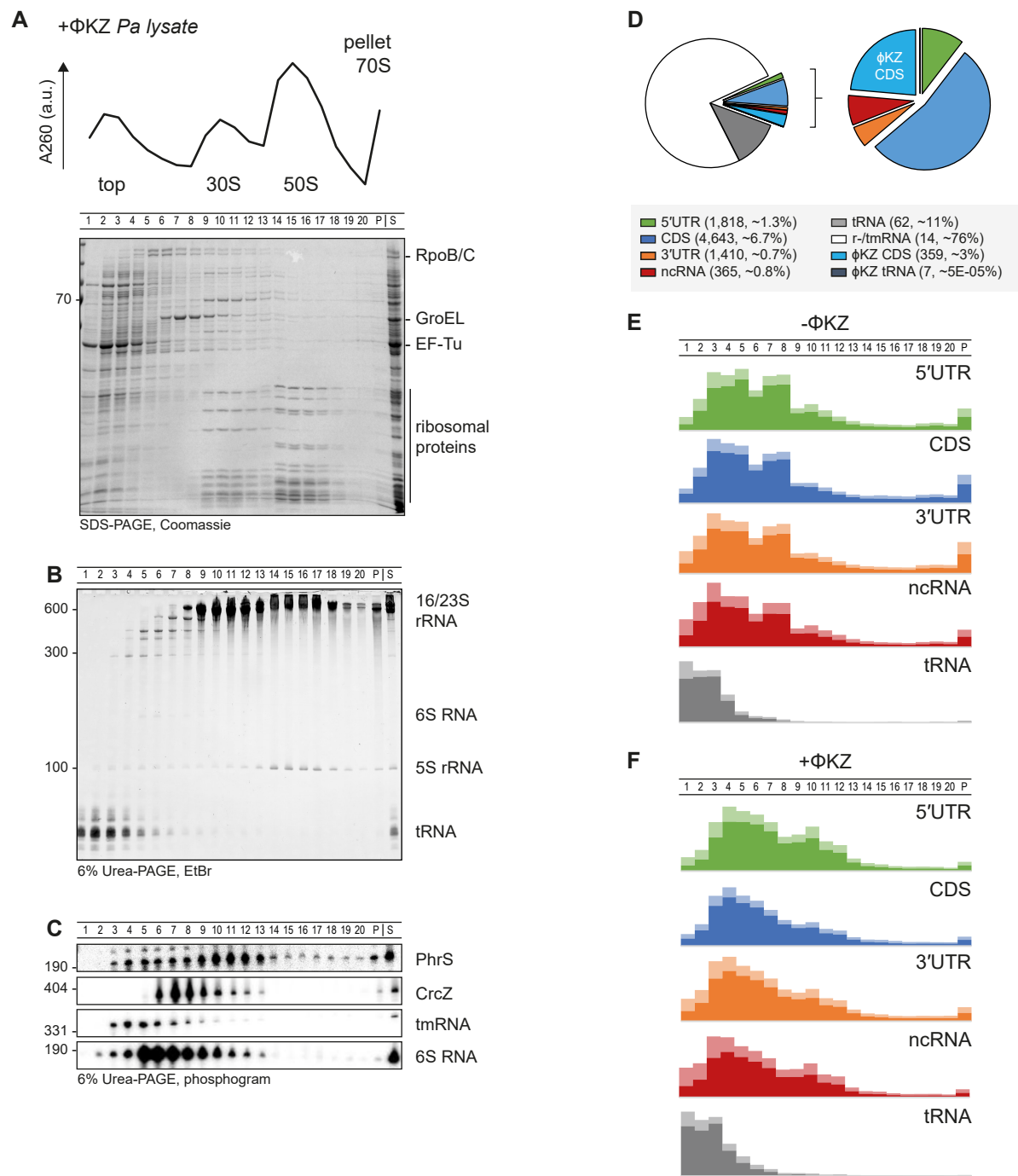

Figure S5

G

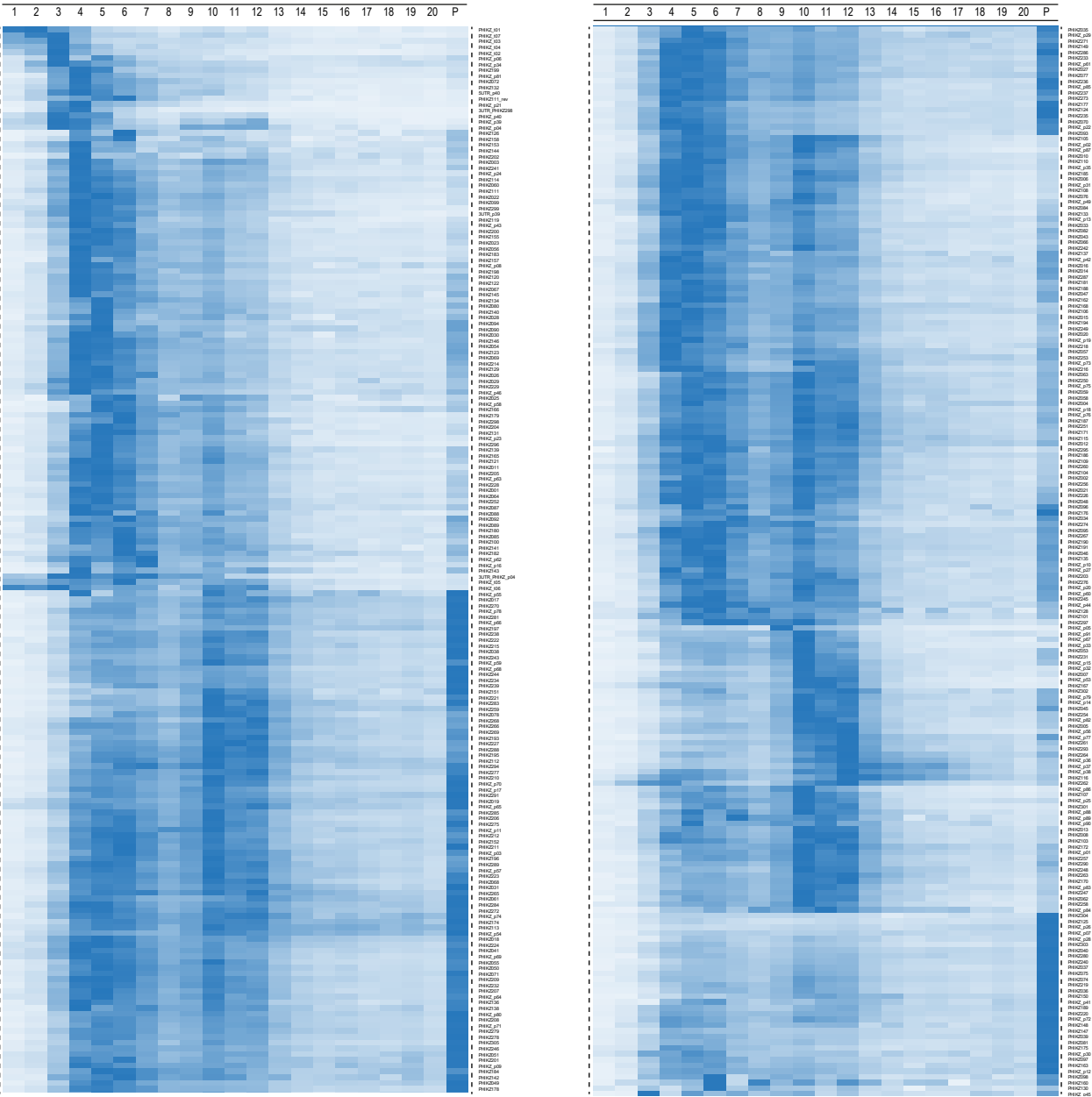

Supplement: FIG S5 [file mbio.03454-20-sf005.pdf]

Figure S7

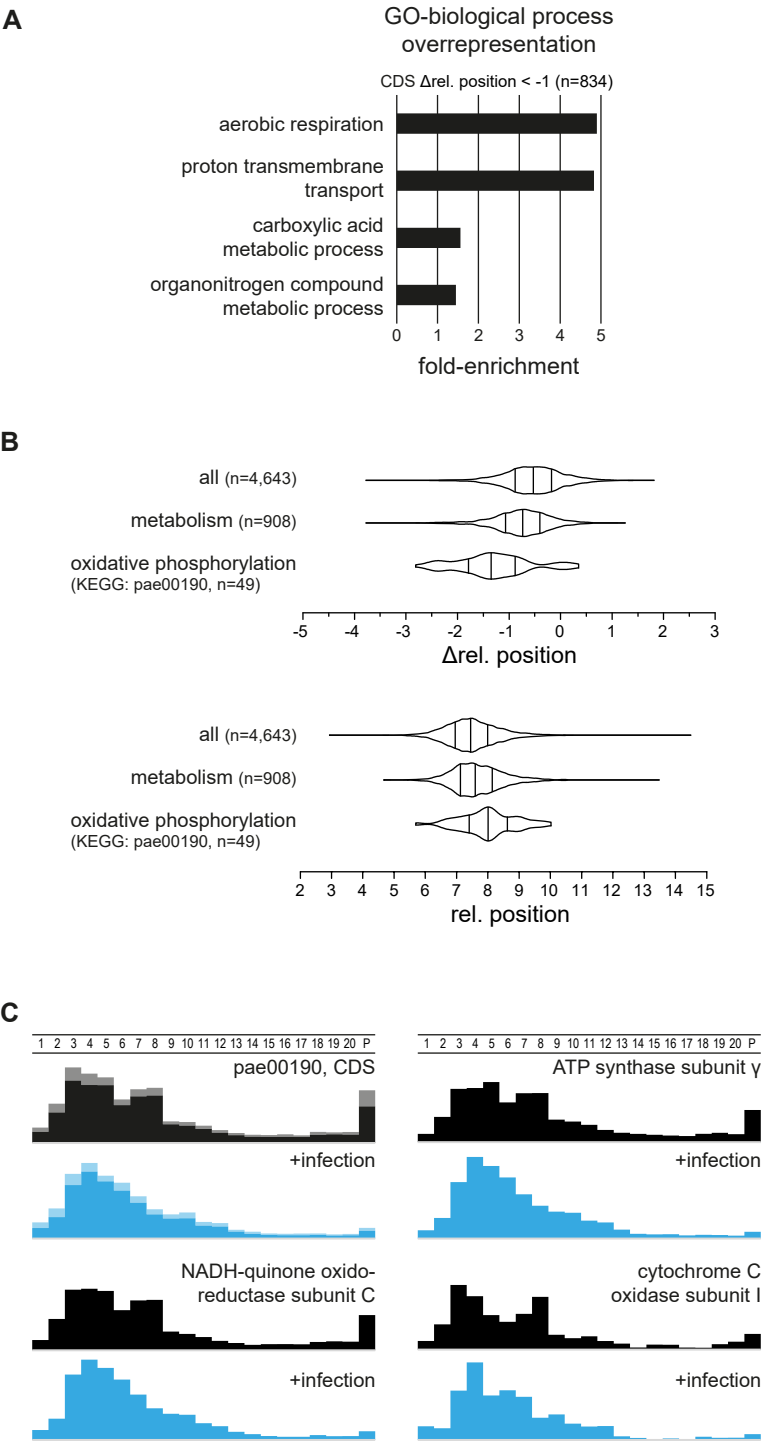

Supplement: FIG S7 [file mbio.03454-20-sf007.pdf]

Figure S8

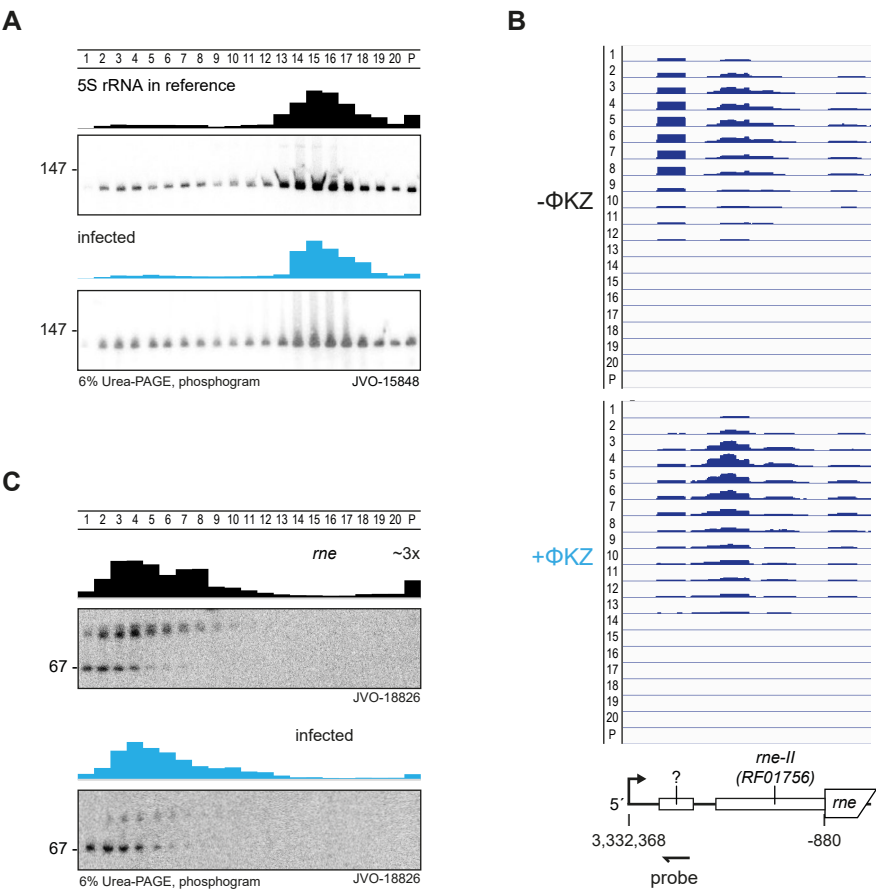

Supplement: FIG S8 [file mbio.03454-20-sf008.pdf]
