# Supplementary material for: A Grad-seq View of RNA and Protein Complexes in Pseudomonas aeruginosa under Standard and Bacteriophage Predation Conditions
Source: mBio. 2021 Feb 9;12(1):e03454-20. doi: 10.1128/mBio.03454-20 (PMC8545117; doi:10.1128/mBio.03454-20)

Figure S6

A

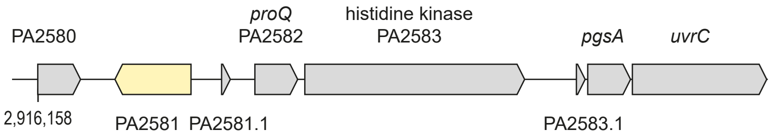

B

FinO-domain (PF04352)

```
putida F1        MGFEQLAELRDRLRAEKGQVKAESAKPPKRKSPPQ---AKPREMDPAVAAIWPLQKHFPL   57
PA7              MGFEQLAELRDRLRAQAEQAKPAKSAQSKAKASPGRAAKKREPVDPEVEAIWRLQRHFPL   60
LESB58           MGFEQLAELRDRLRAQAAQAKPAQTKSS------AGRAKKREAVEPGVEAIWRLQRHFPL   54
PAO1             MGFEQLAELRDRLRAQAAQAKPAQTKSS------AGRAKKREAVEPGVEAIWRLQRHFPL   54
UCBPP-PA14       MGFEQLAELRDRLRAQAAQAKPAQTKSS------AGRAKKREAVEPGVEAIWRLQRHFPL   54
conservation     ***************:  *.*  .:              * . ::* * *** **:****
```

important residues in ProQ from *E. coli* (Pandey et al. 2020): K35, G37, Y70, R80, D82

```
putida           AFPVNPAPKVPLKEGIFKDAEQHLELLGLTREQLKLGISTWCRGARYWASMVENAPRLDL   117
PA7              AFPKNPAPKVPLKQGILEDAQQHLESLGITAEQLKQAIATWCQGNRYWSCMVEDAPRLDL   120
LESB58           AFPKSPAAKVPLKQGILQDAQQHLELLGITAEQLKQAIATWCQGSRYWSCMVEDAPRLDL   114
PAO1             AFPKSPAAKVPLKQGILQDAQQHLELLGITAEQLKQAIATWCQGSRYWSCMVEDAPRLDL   114
UCBPP-PA14       AFPKSPAAKVPLKQGILQDAQQHLELLGITAEQLKQAIATWCQGSRYWSCMVEDAPRLDL   114
conservation     *** .** *****:**::**:**** **:* **** .*:***:* ***:.***:******
```

G85

```
putida           NGQAAGTVTAAQALHAKQQAARQRSQDRRNRAKSKAQAQAQAPAPAAD-TATVQSTD---   173
PA7              QGQVAGKVTAEQAVYAKRQASRRQRDQMREKRAKRARAATESAADKLESDA-----DAQP   175
LESB58           QGQVAGKVTAEQAVYARRQASRRQREQMREKRAKRAQADSEAAAATEAPTPEASATEASP   174
PAO1             QGQVAGKVTAEQAVYARRQASRRQREQMREKRAKRAQAGGEAPAATEAPTPEAPATEASP   174
UCBPP-PA14       QGQVAGKVTAEQAVYARRQASRRQREQMREKRAKRAQAGSEAPAATEAPTPEAPATEASP   174
conservation     :**.**.*** **::*::**:*:: :: *::  .:*:*  :: *            :

putida           -----    173
PA7              DSSAF    180
LESB58           EAN--    177
PAO1             EAN--    177
UCBPP-PA14       EAN--    177
```

C

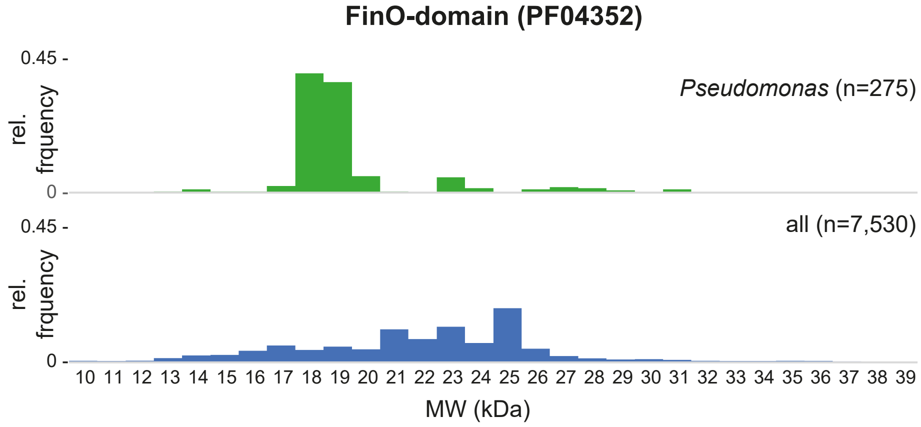

Supplement: FIG S6 [file mbio.03454-20-sf006.pdf]
